# Oral Toxicity of Okadaic Acid in Mice: Study of Lethality, Organ Damage, Distribution and Effects on Detoxifying Gene Expression

**DOI:** 10.3390/toxins5112093

**Published:** 2013-11-08

**Authors:** Andres C. Vieira, Juan A. Rubiolo, Henar López-Alonso, José Manuel Cifuentes, Amparo Alfonso, Roberto Bermúdez, Paz Otero, Mercedes R. Vieytes, Félix V. Vega, Luis M. Botana

**Affiliations:** 1 Departamento de Farmacología, Facultad de Veterinaria, Universidad de Santiago de Compostela, Lugo 27002, Spain; E-Mails: andres.crespo@usc.es (A.C.V.); ja.rubiolo@usc.es (J.A.R.); henar.lopez@usc.es (H.L.-A.); amparo.alfonso@usc.es (A.A.); mariapaz.otero@usc.es (P.O.); 2 Departamento de Fisiología, Facultad de Veterinaria, Universidad de Santiago de Compostela, Lugo 27002, Spain; E-Mails: mmercedes.rodriguez@usc.es (M.R.V.); felixvictor.vega@usc.es (T.V.V.); 3 Departamento de Anatomía, Facultad de Veterinaria, Universidad de Santiago de Compostela, Lugo 27002, Spain; E-Mails: m.cifuentes@usc.es (J.M.C.); roberto.bermudez@usc.es (R.B.)

**Keywords:** okadaic acid, histopathology, diarrhea, immunohistochemistry, liver, superoxide dismutase 1, catalase, quantitative PCR, mice

## Abstract

*In vivo*, after administration by gavage to mice and rats, okadaic acid has been reported to produce lesions in liver, small intestine and forestomach. Because several reports differ in the damage detected in different organs, and on okadaic acid distribution after consumption, we determined the toxicity of this compound after oral administration to mice. After 24 hours, histopathological examination showed necrotic foci and lipid vacuoles in the livers of intoxicated animals. By immunohistochemical analysis, we detected this toxin in the liver and kidneys of intoxicated animals. Okadaic acid induces oxidative stress and can be activated *in vitro* into reactive compounds by the post-mitochondrial S9 fraction, so we studied the okadaic effect on the gene expression of antioxidant and phase II detoxifying enzymes in liver. We observed a downregulation in the expression of these enzymes and a reduction of protein expression of catalase and superoxide dismutase 1 in intoxicated animals.

## 1. Introduction

Okadaic acid (OA) is one of the toxins responsible for the human intoxication known as diarrhetic shellfish poisoning (DSP), which appears after the consumption of contaminated shellfish [1] This polyether toxin is produced by dinoflagellates of the genera Dinophysis or Prorocentrum [2]. Together with its analogs, dinophysis toxin-1 (DTX-1, 35-*R*-methyl OA), -2 (DTX-2, 31-demethyl-35-*S*-methyl OA) and -3 (DTX-3, esterified forms of the parent toxins, DTX-1 and -2), it accumulates in shellfish as a result of filter feeding and ultimately causes human poisoning after contaminated shellfish consumption [3]. OA, DTX-1 and -2 are potent inhibitors of 1, 2a and 3 serine/threonine protein phosphatases (PP1, PP2A and PP3) [4], PP2A being the enzyme most strongly inhibited by these toxins [5,6]. Phosphatase inhibition by OA on intestinal cells has been reported to increase paracellular permeability, causing symptoms like diarrhea after oral intoxication [7]. Phosphatase inhibition and the tumor necrosis factor-α (TNF-α) induction have been shown to play an important role in the biochemical process of tumor promotion in several organs, including skin, liver and stomach [4]. 

Oral administration of OA showed highly variable effects: in rodents, death by orally administered OA has been reported at various doses, ranging from 400 to 2000 µg/kg. Death was preceded by liver, small intestine and forestomach damage [8,9,10], showing the appearance of dark areas in liver and an increase of transaminases (aspartate- and alanine-aminotransferase) in blood [9,10]. Also, an apoptosis increase in the liver of mice dosed with OA at similar concentrations has been reported [8]. On the other hand, other authors did not report liver damage after OA intoxication in mice at concentrations ranging from 75 to 400 µg/kg [11], or in rats at concentrations ranging from 555 to 830 µg/kg [12]. Some research groups have observed small intestine damage after oral administration of OA [9,11,13,14], while other groups did not report this effect [8,10,15].

When radioactive OA was administered at concentrations that produced mild intoxication in mice (50 µg/kg), the highest concentrations of the toxin were found in intestine, liver and stomach after 24 hours of administration. The total amount of OA found in tissues, for the dose previously stated, was low compared to the amount excreted in urine and feces [16]. By immunohistochemical analysis, Le Hégarat *et al*. [8] detected this toxin in duodenum, ileum and liver, 24 hours after administration by gavage, while Ito *et al*. [11] detected it in lungs, heart, liver, kidneys, stomach, small intestine, cecum and large intestine 24 hours after administration, and up to 4 weeks in the intestine. 

Aune *et al.* [13], in a study of synergistic effects of OA and azaspiracids (a new phycotoxin, recently discovered), showed that the OA administration did not produce diarrhea, with high levels of the toxin found in tissues from the GI tract. Lower levels of toxin were also found in liver, kidneys, spleen, and lungs. No pathological changes could be observed. While intestinal damage is hard to be seen due to the characteristics of the GI tract, Wang *et al.* [17], showed ultrastructural damage in intestinal microvilli 3 hours after OA administration, with a total recovery after 24 hours.

Due to the variability observed in mortality, in addition to the organs affected and tissue distribution, we decided to assay the oral toxicity of OA in mice. Tissue damage after toxin treatment was determined by histopathological examination of intestine, liver, kidneys, heart, lungs and brain, while OA tissue distribution was determined by immunohistochemistry. 

OA can be metabolized and activated *in vitro* into reactive compounds that induce chromosome damage, by the post-mitochondrial S9 fraction [18]. Metabolites obtained by phase I detoxification enzymes are only slightly less potent inhibitors of serine threonine protein phosphatase 2A (PP2A) when compared to OA [19,20]. Additionally, the tumor promoting activity of OA and functionally related compounds was proposed to be due to a pro-oxidant activity of this toxin [21] Taking into account these observations, and considering that OA circulates through the enterohepatic cycle, the gene expression of antioxidant and phase II enzymes was determined in the liver of both control and OA-treated mice in order to establish if it affected the expression of these detoxifying systems. Based on the results obtained, the expression of superoxide dismutase-1 and catalase was also determined.

## 2. Results

Diarrhea was almost instantaneous in intoxicated animals, and OA could be detected in the feces at doses of 700 μg/kg and 1000 μg/kg after the first hour post-intoxication (Table 1). Of the three OA concentrations tested, only the highest one (1000 μg/kg) was lethal in approximately 30% of the cases. At 700 μg/kg, it was only lethal to 1 of 13 intoxicated animals (Table 2). All animals suffered diarrhea after OA intoxication, while those intoxicated with the lowest concentration (500 μg/kg), quickly recovered and had no symptoms (persistent diarrhea) after 24 hours of OA administration. 

**Table 1:** OA determination in feces and blood of intoxicated mice after one hour.

OA dose (μg/kg)	Number of animals	OA excreted by feces (ng)	OA still in the organism (%)	OA in blood (ng/μL)
Control	1	n/d	0	n/d
500	4	n/d	100	n/d
700	4	68.34 ± 38.058	>99	0.113 ± 0.047
1000	4	90.565 ± 23.977	>99	0.232 ± 0.104

Notes: LOD feces: 0.00587 ng/mg; LOD blood: 0.30 ng/mL.

Even so, more than 99% of the OA ingested by the animals remained in their bodies, and it could also be detected in blood (Table 1).

**Table 2:** Experimental design.

Treatments	4 Experiments												Combined results			
	Diarrhea				Mortality				Symptoms after 24 hours							
	Exp. 1	Exp. 2	Exp. 3	Exp. 4	Exp. 1	Exp. 2	Exp. 3	Exp. 4	Exp. 1	Exp. 2	Exp. 3	Exp. 4	Total Animals	Diarrhea	Mortality (%)	Symptoms after 24 hours
Control (vehicle)	**-**	**-**	**-**	**-**	**0/3**	**0/3**	**0/3**	**0/1**	**–**	**–**	**–**	**–**	**10**	**–**	**0.0%**	**–**
OA 500 µg/kg	**+**	**+**	**+**	**+**	**0/3**	**0/3**	**0/3**	**0/4**	**–**	**–**	**–**	**–**	**13**	**+**	**0.00%**	**–**
OA 700 µg/kg	**+**	**+**	**+**	**+**	**1/3**	**0/3**	**0/3**	**0/4**	**+**	**+**	**+**	**+**	**13**	**+**	**7.70%**	**+**
OA 1000 µg/kg	**+**	**+**	**+**	**+**	**1/3**	**1/3**	**1/3**	**1/4**	**+**	**+**	**+**	**+**	**13**	**+**	**30.80%**	**+**

Note: Exp.: Experiment.

**Figure 1:**
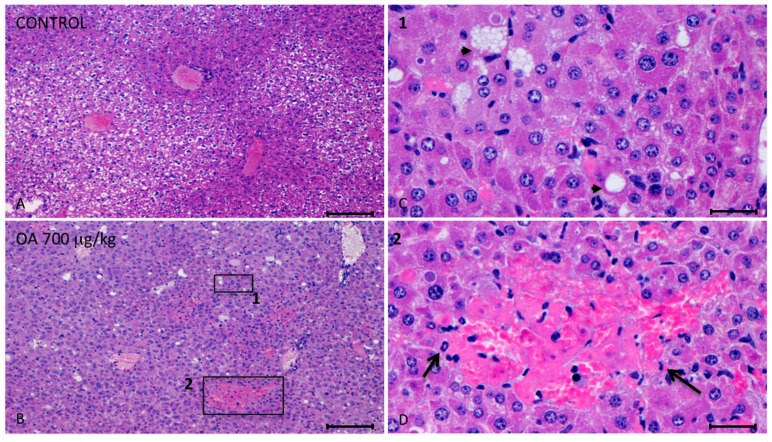
Photomicrographs of liver sections from both control and treated animals. (**A**–**D**) Photomicrographs of liver sections from control (**A**) and 700 μg/kg OA-intoxicated mice (**B**–**D**) showing necrotic areas in liver of treated animals (**B**). (**C**) Amplified area of photomicrograph B (1). Neighboring hepatocytes to the necrotic lesions showed cellular swelling, lipid vacuoles of different sizes (arrow heads) and either pleomorphic or pyknotic nuclei. (**D**) Amplified area of photomicrograph B (2). Multifocal aggregates of necrotic hepatocytes randomly located with the consequent dilation of the sinusoids, and polymorphonuclear inflammatory infiltrates (arrows) could be observed. Scale bars, 200 μm (**A** and **B**) and 20 μm (**C** and **D**).

Twenty-four hours after intoxication, animals were sacrificed and samples of the liver, kidneys, brain, heart, lungs and small intestines were fixed in Bouin’s solution. Fixed slices of the different organs were stained with hematoxylin/eosin and analyzed. We observed important injuries in the liver of 700 μg/kg OA-treated animals (Figure 1).

**Figure 2:**
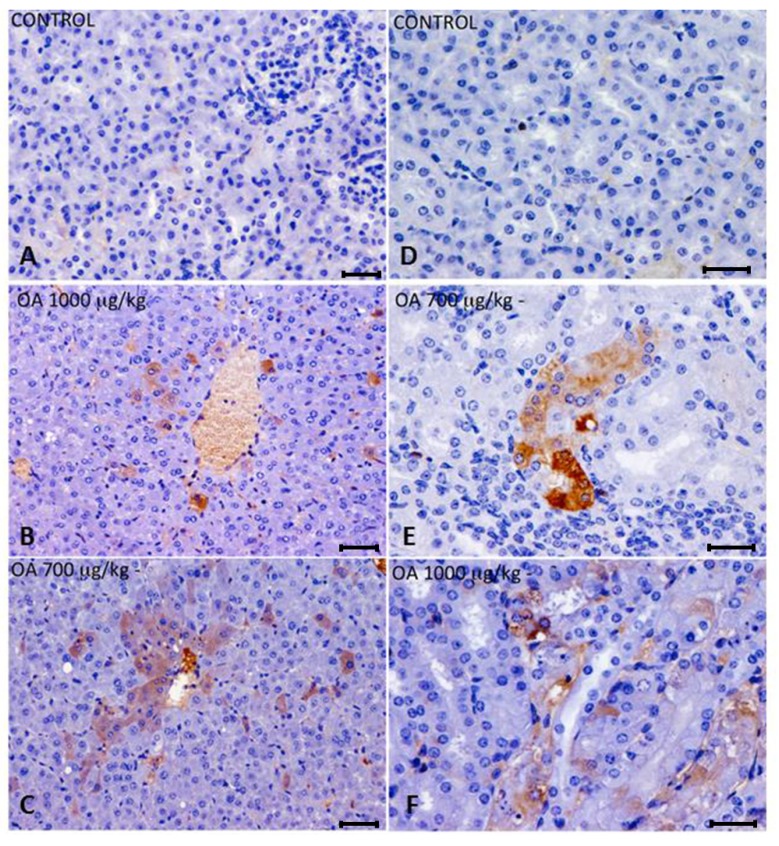
(**A**–**C**) Immunohistochemistry of hepatic sections of control and OA-intoxicated mice. (**A**) Control liver section. (**B**,**C**) OA detected was distributed throughout the liver parenchyma and also concentrated around centrilobular areas in 1000 µg/kg (**B**) and 700 µg/kg (**C**) treated specimens. (**D**–**F**) Immunohistochemistry of kidney sections of control and OA-intoxicated mice. (**D**) Control kidney section.(**E**) 700 µg/kg treated specimens, showing strong immunoreactivity in proximal convoluted tubules. (**F**) 1000 µ/kg treated mice showing that immunoreactivity against OA was distributed in a scattered manner in proximal tubules. Cytoplasm of tubular epithelial cells was vacuolated and nuclear changes were evident.Scale bars 60 μm (**A**–**C**) and 20 μm (**D**–**F**).

These lesions presented multifocal aggregates of necrotic hepatocytes randomly located, with the consequent dilation and congestion of sinusoids in these areas. Neighboring hepatocytes showed cellular swelling, lipid vacuoles of different sizes and either pleomorphic or pyknotic nuclei. Scant polymorphonuclear inflammatory infiltrates could be seen (Figure 1). The cytoplasm of tubular epithelial cells was vacuolated and nuclear changes were present, pointing out necrosis of the tubular epithelium, coinciding with immunoreactivity of kidney cells (Figure 2F).

No histopathological damage was observed in any of the other organs (lungs, heart, intestine and brain) analyzed by this technique at all OA concentrations tested. 

OA distribution was studied by immunohistochemistry in intoxicated and control animals. We detected OA in livers and kidneys of 700 and 1000 μg/kg treated animals, while it was not detected in 500 μg/kg treated or control mice. In liver, immunoreactive cells against the OA antibody were randomly distributed throughout the liver parenchyma, and were also observed around the centrilobular areas (Figure 2A–C). An increase in lipidoses was also perceived (data not shown).

Kidneys of animals treated with 700 μg/kg OA showed strong immunoreactivity in proximal convoluted tubules, although no evident morphological changes were noticed. In the case of animals treated with 1000 μg/kg OA, the immunoreactivity was distributed in a scattered manner in proximal tubules, coinciding with histopathological observations after hematoxylin and eosin (H and E) staining (Figure 2D–F). 

**Figure 3:**
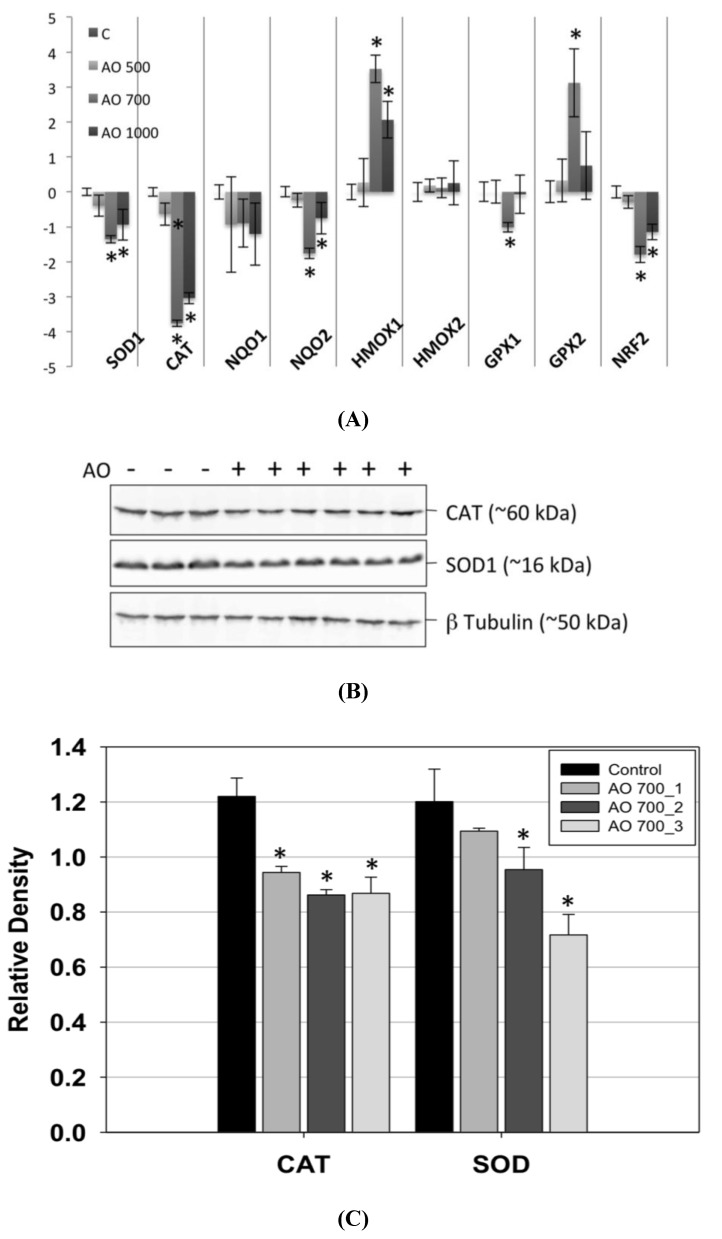
Real-time polymerase chain reaction (PCR) of liver mRNA, from control and OA-treated mice. (**A**) A decrease in the expression of SOD1 and CAT was observed. While the levels of NQO2 mRNA fell in livers of OA-treated animals, an increase in the expression of HMOX2 and GPX2 was observed. There was also a decrease in the expression of the transcription factor Nrf2 in OA-intoxicated animals. (**B**) Western blots of CAT, SOD1 and β tubulin (loading control) of control (−) and OA-treated mice (+). (**C**) Densitometry analysis of the Western blot results showing downregulation of CAT and SOD1 in animals intoxicated with 700 µg/kg OA. * Significant differences with respective controls (*p* < 0.05, *n* = 2).

After studying the detoxifying enzyme gene expression profile by quantitative PCR in control and OA-treated mice, we observed a decrease in the expression of the mRNAs coding for superoxide dismutase-1 (SOD1) and catalase (CAT). The CAT reduction doubled that of SOD1. No NADPH oxydoreductase 1 (NQO1) variation was observed in OA-treated animals when compared to controls. On the other hand, a significant decrease in the NADPH oxydoreductase 2 (NQO2) expression was observed. Heme oxygenase 1 (HMOX1) was increased in the livers of intoxicated animals, whereas no change was observed in Heme oxygenase 2 (HMOX2), in agreement with previous *in vitro* studies [22], where mRNA expression of HMOX1 was induced by OA in cultured hepatocytes. In the case of glutathione peroxidase 1 and 2 (GPX1, 2), only a small increase in the expression of the GPX2 mRNA was detected. Since the antioxidant enzymes transcription is activated by the transcription factor Nrf2, its expression was analyzed. We observed a decrease of nuclear factor (erythroid-derived 2)-like 2 (Nrf2) mRNA in animals intoxicated with 700 and 1000 μg/kg OA (Figure 3A).

Western blot analysis of the expression of CAT and SOD1 in control and in 700 μg/kg OA-intoxicated mice showed a decrease of these enzymes in the livers of intoxicated animals (Figure 3B). The decrease of expression of CAT was of a similar magnitude in all animals analyzed, whereas it had a higher variation for SOD1 (Figure 3C).

**Figure 4:**
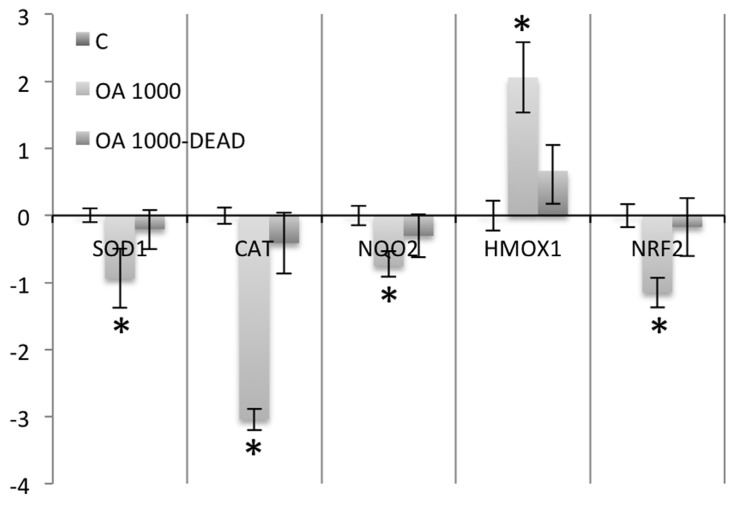
Real-time PCR comparing SOD1, CAT, NQO2, HMOX1 and NRF2 expression in nine mice that survived 1000 μg/kg OA-intoxication (24 hours) with three animals that died early after intoxication (between the hours 3 and 5) with the same dose of toxin.

Conversely to what was observed in intoxicated animals that survived the intoxication up to 24 hours, the study of the antioxidant enzyme expression in animals that died early after intoxication (between 3 and 5 hours) showed that the expression of the enzymes studied was not significantly altered when compared to control cells (Figure 4).

## 3. Discussion

After oral intoxication with OA and histopathological examination of all tissues analyzed, we observed mainly liver damage in some of the intoxicated mice. Injuries were manifested as necrotic foci that appeared in the perivascular regions. Kidney damage was also reported by H and E and immunuhistochemical methods, although injuries were less prominent. When other tissues were examined, no damage was detected at the three OA concentrations tested. Although other authors have reported small [9,12,13,17,23] and large intestine injuries [11] in mice and rats orally intoxicated with various doses of OA ranging from 80 to 2000 μg/kg, we did not observe any. Also, no damage was observed in lungs, heart, or brain of intoxicated animals. 

Diarrhea appeared almost instantly after OA administration, being detected even one hour after treatment. The rapid excretion of part of the OA indicates that a fraction of the toxin ingested by the animals is not absorbed but instantly eliminated. This is in agreement with results obtained by other authors indicating that after 12 hours, the toxin concentration in different organs is low compared to the amount excreted in the urine and feces [16]. Even though the animals intoxicated with 1000 μg/kg OA appeared to excrete more OA in their feces after one hour of administration, the differences observed were not significant. It is tempting to speculate that at the higher concentration tested, the appearance of diarrhea is so rapid that a significant fraction of the OA is excreted from the organism and is not absorbed. Further experiments are planned to more accurately determine if the higher excretion of toxin is responsible for the lower effects observed in this work for the 1000 µg/kg concentration compared to the 700 μg/kg concentration.

OA was only detected in the liver and kidneys after 24 hours of administration. The toxin was detected in the organs of animals intoxicated with 700 and 1000 μg/kg OA but not in those intoxicated with 500 μg/kg. Matias *et al*. [16], using OA, reported that it was detected in every organ 24 hours after oral administration. Even though the method used in this work for OA detection is less sensitive than radiation-based methods, it detected OA by immunostaining, avoiding the possibility of detecting extensively metabolized forms of the toxin. Other authors employed immunostaining to detect OA after oral administration [8,11]. While detecting OA in the liver, they observed no liver damage using up to 250 μg/kg OA. The higher OA concentrations chosen in this work could explain the liver damage we have observed.

Gene expression of hepatic antioxidant and detoxifying enzymes was affected by OA since we could observe a downregulation in the expression of SOD1 and CAT mRNA in OA-treated animals. When the protein-relative concentrations of these two enzymes were analyzed, both were decreased in animals treated with 700 μg/kg OA. The expression of the transcription factor Nrf2, that regulates their expression, was decreased likely as a consequence of phosphorylation cascades activated after PP2A inhibition by the toxin. Whereas other authors have described a lower CAT activity in cultured cells exposed to OA [24], to our knowledge, this is the first time that this effect is described *in vivo*, thus indicating that part of the liver damage produced by the OA could be due to a decrease in the antioxidant capacity of this organ, with the consequent increase of reactive oxygen species. Also, this would render the liver susceptible to damage induced by other toxins, capable of producing oxidative stress that could be present at the time of intoxication with OA. The important role of this toxin in the induction of oxidative stress has been reported by several *in vitro* [25,26] and *in vivo* studies [27,28]. Besides antioxidant enzymes, the expression of the phase II detoxifying enzymes NQO2 and HMOX1 was affected. NQO1 expression was not altered significantly, but it has to be noted that the liver preferentially expresses the NQO2 isoform of the enzyme [29]. The gene coding for this enzyme has an antioxidant response element (ARE) in its promoter [29], indicating that the decrease in Nrf2 expression could also affect NQO2 expression.

When the antioxidant and phase II enzyme expression was analyzed in the animals that died early after intoxication, no significant variation was observed when compared to controls. Thus, in order to affect the expression of these enzymes in the liver, OA has to circulate for a long time in the organism after toxin exposure.

## 4. Experimental Section

### 4.1. Okadaic Acid

The okadaic acid (Cifga Laboratories, Lugo, Spain) used for this work was 99% pure and dissolved in ethanol.

### 4.2. Animals and Treatments

**Figure 5:**
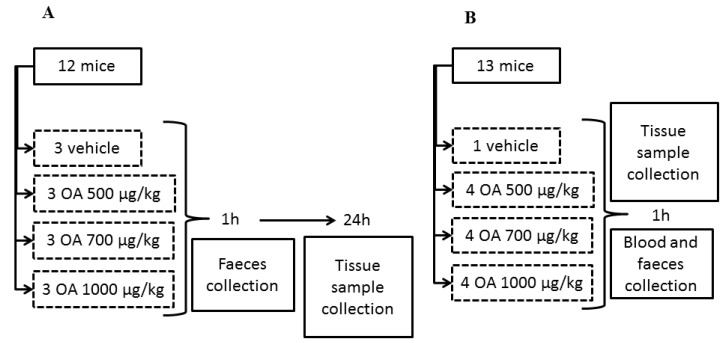
Scheme describing the design of the OA-intoxication experiment and the times when samples for analysis were collected. Scheme A describes an experiment repeated three times, while scheme B describes a unique experiment.

All animal procedures were conducted according to the principles approved by the Institutional Animal Care Committee of the Universidad de Santiago de Compostela. A total of 49 CD-1 female mice (Charles River Inc., Barcelona, Spain) weighing 18–23 g were used. Animals were kept for one week before the experiment at a controlled temperature (23 ± 2 °C) and humidity (60%–70%), as well as fed with a diet for rodents containing 18.5% protein (Harlan^®^). All specimens were singly housed. First, three independent experiments were performed with 12 mice per experiment. Afterward, a fourth experiment was accomplished with 13 animals. All specimens were fed with physiological solution supplemented with glucose for 12 hours before OA administration, in order to have fasted animals at the time of OA treatment. After intoxication, food was available for all the animals. OA was administered by gavage to all experimental specimens at concentrations of 500, 700 and 1000 μg/kg, dissolved in physiological solution containing 2.5% ethanol. Control animals were treated with physiological solution containing 2.5% ethanol (Figure 5).

Samples of feces were collected one hour after toxin administration in all four groups. Surviving specimens of the three initial groups were sacrificed after 24 hours in a carbon dioxide chamber and samples of small intestine, liver, kidneys, lungs, heart and brain were fixed with Bouin’s fixative. Other samples of the same organs were conserved in RNAlater^®^ solution (Ambion^®^) or in liquid nitrogen.

Specimens of the fourth group were sacrificed in a carbon dioxide chamber one hour after OA administration and blood and tissue samples were collected (see Figure 1 for a scheme describing the experimental design). Finally, tissue samples of 4 dead animals between 3 and 5 hours post-OA treatment were also collected. These animals died after intoxication with 700 μg/kg (1 animal) and 1000 μg/kg OA (3 animals), during experiments 1, 2 and 3. Samples were collected at the time of death of each animal to avoid degradation of the RNA and organs.

### 4.3. Histopathology and Immunohistochemistry

Chosen internal organs from mice of each group were fixed with Bouin’s fixative. Paraffin-embedded sections of 3 μm thickness from brain, small intestine, large intestine, kidneys, liver, heart and lungs were dried overnight at 50 °C. Sections were stained with hematoxylin-eosin (H and E) to determine tissue damage or alterations.

For the immunohistochemical assays, special microscope slides with a previous silanization treatment were used. Sections were deparaffinized, rehydrated and pretreated for one hour with Dako Real Peroxidase-Blocking Solution (Dako SA, Barcelona, Spain), to block peroxidase activity and prevent non-specific staining. After removal of blocking reagents, sections were rinsed 3 times in PBS supplemented with 0.005% Tween 20 (Panreac Química SAU, Barcelona, Spain). The sections were then incubated overnight with an anti-OA monoclonal antibody (Santa Cruz Biotechnology, CA, USA) diluted 1:15,000 with an antibody solvent from Dako Real Antibody Diluent (Dako SA, Barcelona, Spain). After rinsing 3 times in PBS-Tween 20, the samples were incubated with the secondary antibody solution, Dako Real Envision HRP Rabbit/Mouse (Dako SA, Barcelona, Spain), for 30 min. Finally, the samples were washed 3 times with PBS-Tween 20 and revealed with DAB+ Chromogen (Dako SA, Barcelona, Spain).

### 4.4. LC-MS/MS Analysis

The feces evacuated by control and intoxicated animals were collected after the first hour of administration. At this time, blood was also obtained from each animal. The OA present in both was determined by liquid chromatography coupled to a mass detector (method M3) as described elsewhere [30].

Briefly, the LC-MS/MS equipment is a combination of HPLC plus mass detector. The HPLC system, from Shimadzu (Kyoto, Japan), consists of two pumps (LC-10ADvp), autoinjector (SIL-10ADvp) with refrigerated rack, degasser (DGU-14A), column oven (CTO-10ACvp) and system controller (SCL-10Avp). This system is coupled with a QTRAP LC/MS/MS system from Applied Biosystems, (USA), which integrate a hybrid quadrupole-linear ion trap mass spectrometer equipped with an ESI source. The nitrogen generator is a Nitrocraft NCLC/MS from Air Liquide (Spain).

The column used for toxins separations was a 2 mm × 50 mm BDS-Hypersil-C8 analytical column with a particle size of 3 µm and a 10 mm × 2.1 mm guard cartridge from Thermo (Waltham, MA, USA). The temperature was set at 25 °C. The composition of the mobile phase was: water (A) and ACN/water (95:5) (B), both containing 50 mM formic acid and 2 mM ammonium formate. Chromatographic separation was performed by gradient elution: starting with 30%–90% B for 8 min, then 90% B was held for 3 min and reduced afterwards to 30% B over 0.5 min and held for 5.5 min until the next run. The mobile phase flow rate was 0.2 mL/min and the injection volume was 5 µL. The mass spectrometer was operated in multiple reaction monitoring (MRM) detecting in negative mode analyzing two product ions per compound: DTX-1 (m/z 817.5 > 255.5, m/z 817.5 > 113.5), OA and DTX-2 (m/z 803.5 > 255.5, m/z 803.5 > 113.5).

### 4.5. RNA Extraction, Integrity Determination and Reverse Transcription

Tissue samples corresponding to the different organs maintained in RNAlater were homogenized in pureZol^TM^ RNA isolation reagent (BIO-RAD) and RNA was isolated following the manufacturer’s instructions. The concentration of the purified RNA was determined spectrophotometrically with a NanoDrop 2000 (Thermo Fisher Scientific Inc., Madrid, Spain) and its integrity was confirmed by denaturing agarose electrophoresis. Samples with 28S rRNA:18S rRNA ratio, determined by densitometry analysis, below 1.7 were discarded. Four micrograms of RNA from each sample were treated with DNAse (Thermo Fisher Scientific Inc., Madrid, Spain). After DNAse treatment, 3 μg of DNA-free RNA corresponding to the different samples to be analyzed was reverse transcribed using an oligo-dT, for total mRNA reverse transcription, with a RevertAid^TM^ M-MuLV Reverse Transcriptase (Thermo Fisher Scientific Inc., Madrid, Spain), following the manufacturer’s instructions.

### 4.6. Quantitative PCR

The cDNA was amplified with specific primers (Table 3) for catalase (CAT), superoxide dismutase-1 (SOD1), heme oxygenase 1 and 2 (HMOX1, 2), NADPH oxydoreductase 1 and 2 (NQO1, 2), glutathione peroxidase 1 and 2 (GPX1, 2) and the transcription factor nuclear factor (erythroid-derived 2)-like 2 (Nrf2) an Applied Biosystems StepOne real-time PCR system with a FastStart^TM^ Universal SYBR Green Master kit (Roche). Cyclophilin (peptidylprolyl isomerase, PPIA) was used as housekeeper (endogenous control) for normalization. Primers were designed using the Primer Express 3.0 Software (Applied Biosystems). Reference mRNA sequences were obtained from the NCBI Nucleotide Database; the accession numbers of each cDNA target are detailed in Table 3. The Stepone software (Applied Biosystems) was used to calculate the fold change and standard deviation of each cDNA from OA-treated animals with respect to control animals. Calibrator ΔC_T_ values were calculated as the difference between the C_T_ (threshold cycle) value of the endogenous control and target. This ΔC_T_ for the calibrator (cDNA from control animals) was compared with the ΔC_T_ value of each of the unknown cDNA samples (from treated animals). The differences between these values resulted in the ΔΔC_T_ and the standard deviation of the ΔΔC_T_ for each cDNA. The relative fold expression (RQ) was determined by 2^−^^ΔΔCT^, and was plotted as base 2 logarithms of the RQ. The error bars display the calculated maximum (RQMax) and minimum (RQMin) expression levels that represent standard deviation of the mean expression level (RQ value). Each treatment was analyzed in triplicate and three experiments were done. 

**Table 3:** Primer sequences used to amplify antioxidant and phase II enzymes in liver and kidney of control and OA-treated mice.

TARGET cDNA	GeneBank accession	PRIMER SEQUENCE
**CAT-catalase**	**NM_009804.2**	5' GCTGAGAAGCCTAAGAACGCAAT 3'
		5' CCCTTCGCAGCCATGTG 3'
**SOD1-superoxide dismutase 1, soluble**	**NM_011434.1**	5' CCAGTGCAGGACCTCATTTTAAT 3'
		5' TCTCCAACATGCCTCTCTTCATC 3'
**NQO1-NAD(P)H dehydrogenase, quinone 1**	**NM_008706.5**	5' GGTTTACAGCATTGGCCACACT 3'
		5' TTCCAGACGTTTCTTCCATCCT 3'
**NQO2-NAD(P)H dehydrogenase, quinone 2**	**NM_020282.3**	5' GTTCACACTCAGCTTCTCTTGCA 3'
		5' GGACACAATACCCAAAGGGAAA 3'
**HMOX1-heme oxygenase (decycling) 1**	**NM_010442.2**	5' CCTCACTGGCAGGAAATCATC 3'
		5' CCTCGTGGAGACGCTTTACATA 3'
**HMOX2-heme oxygenase (decycling) 2**	**NM_010443.2**	5' CCAATTCTACCTGTTTGAGCATGT 3'
		5' AAATTCAGGTCCAAGGCATTCA 3'
**GPX1-glutathione peroxidase 1**	**NM_008160.5**	5' CCCGTGCGCAGGTACAG 3'
		5' CAGCAGGGTTTCTATGTCAGGTT 3'
**GPX2-glutathione peroxidase 2 (gastrointestinal)**	**NM_030677.2**	5' GCTGCCCTACCCTTATGATGAC 3'
		5' GCACGGGACTCCATATGATGA 3'
**Nrf2-nuclear factor erythroid-derived 2-like 2**	**NM_010902.3**	5'-CCGAGATATACGCAGGAGAGGTA-3'
		5'-GCTCGACAATGTTCTCCAGCTT-3'
**PPIA-peptidylprolyl isomerase A (cyclophilin A)**	**NM_008907.1**	5' CAAATGCTGGACCAAACACAA 3'
		5' GCCATCCAGCCATTCAGTCT 3'

### 4.7. Western Blot

Excised livers frozen in liquid nitrogen were ground using a mortar. The ground tissue was homogenized in RIPA lysis buffer containing a protease inhibitor cocktail (ROCHE). After lysis, samples were centrifuged at 14,000 rpm at 4 °C for 15 min. The soluble proteins were recovered and quantified by infrared spectrometry using the Direct Detect™ Biomolecular Quantitation System (MILLIPORE).

Equal amount of protein for each condition assayed were resolved by SDS-PAGE (12% acrylamide, 70 V, 3 hour). After electrophoresis, proteins were transferred to PVDF membranes for one hour at 100 V. Membranes were blocked overnight with 3% milk and 0.1% Tween 20, and then incubated with the primary antibodies (anti-CAT (SIGMA) 1:3000, anti-SOD1 (SIGMA) 1:1000 and anti-β tubulin (SIGMA) 1:1000) dissolved in blocking solution, for 3 hours at room temperature. After washing 3 times for 10 min with PBS and 0.1% Tween 20, the membranes were incubated with 1:5000 anti-mouse secondary antibody (SIGMA) dissolved in blocking solution for one hour at room temperature. Membranes were washed 3 times for 10 min with PBS and 0.1% Tween 20 and revealed using the SuperSignal^®^ West Pico (Fisher Scientific) for CAT and tubulin, or SuperSignal^®^ West Femto (Fisher Scientific) for SOD1, following the manufacturer instructions.

Images were analyzed using the ImageJ [31] software and the relative density, using tubulin as loading standard, was plotted and is shown in this work.

### 4.8. Statistical Analysis

Differences in CAT and SOD1 expression determined by Western blot were analyzed using SIGMAPLOT^©^. One-way ANOVA was used for comparison of significant differences among groups. Differences between groups were determined by the Holm–Sidak multiple range test. A *p* < 0.05 was considered significant.

## 5. Conclusions

This work shows that OA is detected in liver and in kidneys of mice 24 hours after administration by gavage. The toxin remained undetectable in the small intestine, stomach, lungs and brain. In the liver, necrosis was observed, while OA produced no lesions in the small intestines, stomach, lungs, brain and kidneys. OA induced a decrease in the liver antioxidant and detoxifying capacity by downregulating the expression of the antioxidant enzymes SOD1 and CAT, and the phase II enzyme NQO2.
